# Tissue Engineering of the Corneal Endothelium: A Review of Carrier Materials

**DOI:** 10.3390/jfb4040178

**Published:** 2013-10-22

**Authors:** Juliane Teichmann, Monika Valtink, Mirko Nitschke, Stefan Gramm, Richard H.W. Funk, Katrin Engelmann, Carsten Werner

**Affiliations:** 1 Leibniz Institute of Polymer Research Dresden, Max Bergmann Center of Biomaterials, Institute of Biofunctional Polymer Materials, Hohe Straße 6, Dresden 01069, Germany; E-Mails: teichmann@ipfdd.de (J.T.); nitschke@ipfdd.de (M.N.); gramm@ipfdd.de (S.G.); werner@ipfdd.de (C.W.); 2 Institute of Anatomy, Medical Faculty Carl Gustav Carus, Technische Universität Dresden, Fetscherstraße 74, Dresden 01307, Germany; E-Mail: richard.funk@tu-dresden.de; 3 CRTD/DFG-Center for Regenerative Therapies Dresden—Cluster of Excellence, Fetscherstraße 105, Dresden 01307, Germany; 4 Department of Ophthalmology, Klinikum Chemnitz gGmbH, Flemmingstraße 2, Chemnitz 09116, Germany; E-Mail: k.engelmann@skc.de

**Keywords:** tissue engineering, corneal endothelium, corneal endothelial cell sheets, natural membranes, biological polymers, thermo-responsive polymers, physicochemical properties, biomolecular functionalization

## Abstract

Functional impairment of the human corneal endothelium can lead to corneal blindness. In order to meet the high demand for transplants with an appropriate human corneal endothelial cell density as a prerequisite for corneal function, several tissue engineering techniques have been developed to generate transplantable endothelial cell sheets. These approaches range from the use of natural membranes, biological polymers and biosynthetic material compositions, to completely synthetic materials as matrices for corneal endothelial cell sheet generation. This review gives an overview about currently used materials for the generation of transplantable corneal endothelial cell sheets with a special focus on thermo-responsive polymer coatings.

## 1. Introduction

### 1.1. The Cornea

**Figure 1:**
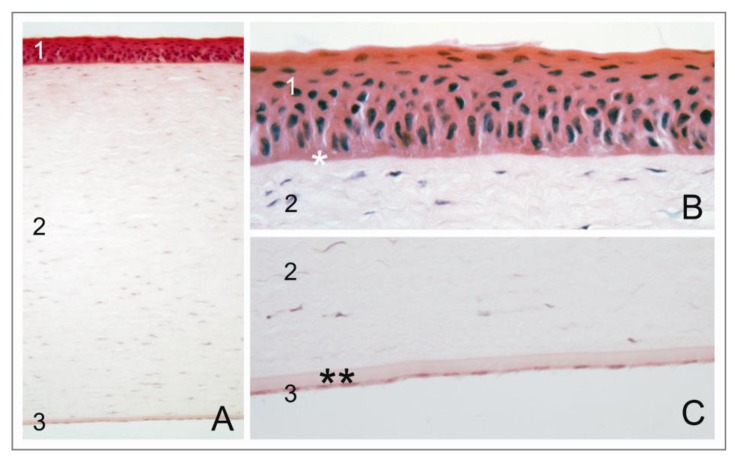
Structure of the human cornea (**A**) The cornea is composed of three cellular layers: (1) epithelium; (2) stroma; and (3) endothelium; (**B**) The epithelial layer resides on Bowman’s membrane (*); and (**C**) the endothelial layer resides on Descemet’s membrane (**).

The cornea is a transparent tissue with a diffractive capacity of about 43 diopters that allows light to enter the eye and evoke a visual sensation in the retina. It is a bradytrophic tissue, meaning that it is avascular and not nourished via the blood system, but subsists on tear film and aqueous humor [1]. The cornea has a dome-shaped structure with a strictly layered architecture (Figure 1) [2,3]. Its average central thickness ranges from 530 µm to 550 µm [4] and increases to an average peripheral thickness of up to 670 µm [5]. The anterior cornea is covered by a stratified squamous epithelium, which produces mucins to retain the tear film. Epithelial progenitor cells reside in niches at the anterior corneal limbus and ensure a continuous regeneration of the epithelium. These limbal stem cells proliferate and migrate centripetally, thereby stratifying and differentiating [6,7]. The basal epithelial cells reside on Bowman’s membrane, a condensed layer comprised of mainly collagen type I that cannot be reproduced once it is destroyed. This membrane is penetrated by sensitive nerve fibers that form a subepithelial plexus in the central cornea [8,9]. The corneal stroma makes up to about 90% of the corneal thickness and is predominantly composed of collagen type I layers. These layers are interspersed with keratocytes that produce mainly proteoglycans and collagen. The collagen fibers are arranged in parallel in lamellae, and the direction of fibers in neighboring lamellae is mostly perpendicular [10,11,12]. Corneal avascularity and the lattice structure of stromal collagen fibers contribute to corneal transparency. Hydration of the corneal stromal plays a major role in maintaining this transparency, as glycosaminoglycan side chains of the proteoglycans, such as keratan sulfate, dermatan sulfate and chondroitin sulfate, exert a high swell pressure due to their water binding capacity. Excessive fluid imbibition by the stroma, usually due to a dysfunction in the corneal hydration regulatory system, can lead to corneal edema with haze or opacification and can eventually result in blindness. The posterior side of the corneal stroma is lined by Descemet’s membrane, the basal membrane of the posterior epithelium of the cornea, the corneal endothelium. Descemet’s membrane can grow up to 10–12 µm in thickness and is mainly comprised of collagens type IV and VIII [11], but does also contain fibronectin, vitronectin and various laminins [13]. This membrane adheres only weakly to the stroma and can be detracted. The corneal endothelium is a monolayer of hexagonal, squamous cells lining the posterior cornea [14]. Contrasting to the anterior epithelium, the posterior corneal endothelium cannot be regenerated, although limited regeneration has been observed in some cases after graft failure in younger patients (own clinical observation), and a recent report suggests that a minor regenerative capacity may exist in the utmost periphery in close proximity to the trabecular meshwork [15]. 

### 1.2. The Corneal Endothelium

With age, central corneal endothelial cell density gradually declines at a rate of 0.5%–0.6% per year [16]. This endothelial cell loss is compensated for by migration and enlargement of neighboring cells, but not by regeneration [14,17], resulting in an endothelium with a reduced number of hexagonal cells (pleomorphism) and greater variations in cell size (polymegathism) [10,18]. Proliferation of corneal endothelial cells *in situ* is suppressed by contact inhibition and by transforming growth factor β2 (TGF-β2), which is secreted into the aqueous humor and prevents entry into the S-phase of the cell cycle [19]. In addition, structural and compositional differences in adult *versus* embryonic Descemet’s membrane may contribute to G1-phase arrest of human corneal endothelial cells (HCEC) [19], e.g., collagen type III may stimulate cell proliferation, but interacts directly with HCEC only during embryonic development. Although cell division is inhibited *in vivo*, the cells are able to proliferate *in vitro*. Primary HCEC were shown to react towards mitogens and growth factors in an age-dependent mode, being more sensitive from younger than from older donors [20,21,22]. It could also be shown that wounding could induce a limited proliferative capacity in peripheral HCEC *in situ* and *ex vivo*, which was, to some extent, dependent on donor age [22]. In this context, it was also demonstrated that p53, a negative cell cycle regulator, is predominantly expressed in the central cornea [23]. These findings led to the hypothesis that the posterior periphery of the cornea may harbor a stem cell niche for corneal endothelial and trabecular meshwork cells, similarly to the anterior limbal stem cell niche of the corneal epithelium [15,24]. This hypothesis is supported by the observation that the stem cell markers, nestin, alkaline phosphatase and telomerase, were found in the posterior corneal periphery in the endothelium and trabecular meshwork around Schwalbe’s line. Upon wounding, additional stem cell markers, Oct-3/4 and Wnt-1, and differentiation markers, Pax-6 and Sox-2, were upregulated in the posterior periphery [15]. Such posterior stem cells might contribute to endothelial regeneration and repair in the periphery.

Corneal endothelial cells form an anatomical and physiological barrier between the corneal stroma and the anterior chamber. They sustain an active transport of fluid at a rate of 3.5–6 µL cm^−2^ h^−1^ from the stroma into the anterior chamber, thereby regulating stromal hydration [10,25]. The so-called “pump-leak” hypothesis describes the mechanisms by which corneal hydration is maintained constant [10,26,27,28]. Tight junctions in the corneal endothelium are required to seal off both the stroma and the anterior chamber. Although the tight junctions are interrupted and the seal is not complete, they control the directed, extracellular diffusion of ions and water from the anterior chamber into the corneal stroma (“leaky” barrier [29]). Intact tight junctions also restrict a lateral diffusion of membrane proteins, such as ion transporters, which are required for an active transport of ions and water from the stroma into the anterior chamber (“pump” function). This delimitation supports the apical-basal polarity of the endothelial cells (“fence” function) and the difference of potential across the endothelial layer, thereby sustaining a local osmotic gradient by preventing an uncontrolled paracellular reflux (“gate” function). Two main theories describe the coaction of ion pumps and ion channels involved in transendothelial transport of ions and water. The theory set up by David M. Maurice [25,30] states that basolaterally localized Na^+^/K^+^-ATPase [10,31] actively sustains an osmotic gradient that drives secondary transporters [32]. A passive flow of water from the stroma into the anterior chamber balances the excess of ions that builds up in the aqueous humor upon endothelial pump action. The group around Jorge Fischbarg postulated a model of electro-osmotic coupling of tight junctions [33,34,35,36]. According to their theory, corneal endothelial cells generate a transepithelial potential by directed ion transport. This electrical field causes a directed movement of counter ions with the consequence that stromal water is drawn into the anterior chamber by a pull effect known as electro-osmosis [34]. 

### 1.3. Corneal Endotheliopathies and Therapy by Donor Cornea Transplantation

Pathological endothelial cell loss can be excessive due to disease or trauma. This pathological cell loss cannot be compensated for, as the proliferative capacity of the corneal endothelium is limited and restricted to the corneal periphery. Corneal function deteriorates irreversibly when the endothelial cell density falls below a critical lower threshold, which is presumed to be of ≈ 500 cells mm^−2^ [37], although it was observed that corneas with a cell density below 500 cells mm^−2^ can be functional, as well. Due to the stroma’s high capacity to absorb fluid, a corneal edema can develop, which impairs corneal transparency and, eventually, results in corneal blindness. Common primary corneal endotheliopathies, for example, posterior polymorphous dystrophy [38], congenital hereditary endothelial dystrophy [39], Fuchs’ endothelial dystrophy [40,41] or pseudophakic bullous keratopathy as a post-surgical complication after cataract extraction [10], become manifest in excrescences and thickening of Descemet’s membrane and, also, endothelial polymegathism and pleomorphism. The pump and barrier function of the endothelium slowly decrease, and finally, the cornea develops an edema. 

About two million people worldwide suffer from corneal blindness. The gold standard to treat corneal blindness is the transplantation of a donor cornea (penetrating keratoplasty). The cornea can easily be transplanted due to its accessibility, avascularity and immune privilege (meaning that it is disconnected from the immune system to protect the tissue from destructive immunologic reactions) [42]. However, it was also observed that the decrease in corneal endothelial cell density after corneal transplantation is higher compared to the physiological rate [43] and can lead to graft failure. Additional post-surgical problems in keratoplasty are immune reactions and the induction of astigmatism that limits visual outcome [44,45,46,47]. In recent years, various alternative treatment approaches were tested experimentally and clinically and are constantly being improved, e.g., lamellar keratoplasty to replace only the diseased layers of the cornea. In (deep) anterior lamellar keratoplasty (DALK), only corneal epithelium and anterior stroma are replaced, while in posterior lamellar keratoplasty (PLK), only endothelium with Descemet’s membrane is replaced (descemetorhexis, according to Melles *et al*. [48]). Since Descemet’s membrane is only weakly adherent to the stroma, it can easily be removed. The endothelial transplant lamella can then be inserted through a small corneoscleral incision and fixated onto the posterior stroma by injecting a small air bubble. Modified versions are Descemet’s stripping (automated) endothelial keratoplasty (DS(A)EK) [49] and Descemet’s membrane (automated) endothelial keratoplasty (DM(A)EK) [48,50,51]. At present, DMEK using manual techniques is preferred by surgeons. The lamellar approach can help to improve early visual acuity by minimizing post-surgical astigmatism and the risk of immune reactions. However, the interface between recipient corneal tissue and the donor lamella can be irregular and induce a haze in the optical path, which limits post-surgical visual outcome [52,53]. Another disadvantage is the risk of graft dislocation and the need for repeated re-injections of air, since the lamellar graft is not sutured into the host tissue [49,51]. Finally, lamellar grafts also experience an accelerated post-surgical endothelial cell loss [54,55]. Despite these disadvantages, lamellar techniques have the potential to replace penetrating keratoplasty as the gold standard for a variety of corneal endothelial diseases [56].

Endothelial cell loss increases shortly after keratoplasty and is higher after lamellar keratoplasty than after penetrating keratoplasty, but relatively normalizes after approximately two years post-surgery [56,57,58,59,60,61]. Due to the observed higher post-surgical endothelial cell loss, only donor corneas with a high endothelial cell density are transplanted. Storage, processing and transport of corneal donor tissue can impair the quality of the corneal endothelium. It has been shown that improving culture protocols can minimize the cell loss in organ cultured donor corneas, e.g., by using a fully defined, serum-free culture medium with enriched formulation instead of the conventionally used Minimal Essential Medium (MEM) [62,63,64]. The use of culture media that are free of animal components has gained increasing importance for clinical applications, such as cultivation and preservation of donor corneas or corneal cells in tissue engineering approaches. For example, the European Tissue and Cells Directive [65] requires the use of humanized (animal component free) media with a defined composition whenever possible [66], because these media consist of defined substances, do not vary in composition or quality between lots and do not bear the risk of contamination with infectious agents. Research data demonstrated that improved serum-free culture media were superior to commonly used serum-supplemented culture media regarding the maintenance of corneal endothelial cell viability and the preservation of a healthy corneal endothelium of donor corneas [62,63,67,68,69,70].

### 1.4. Keratoprostheses

The limited availability of human donor tissue and frequent graft failure have furthered the development of artificial corneal replacement materials, such as keratoprostheses [52,71,72]. Keratoprostheses are synthetic implants designed for replacing the central cornea. The Boston KPro (previously called Dohlman-Doane KPro) combines a synthetic optical part with a biological holding support [73]. The central optical part consists of transparent poly(methyl methacrylate) (PMMA), a porous back plate and a titan ring, which holds a trephined donor cornea [52,71,72]. The AlphaCor^TM^ KPro has a solid, transparent optical part and a porous, opaque fringe, both made of poly(2-hydroxyethyl methacrylate) (PHEMA) [74]. The porous fringe enables recipient corneal stromal fibroblasts to infiltrate the prosthesis, thereby fixating it in the recipient’s eye [75]. Nevertheless, none of the keratoprostheses meets the demands for an ideal artificial transplant with respect to proper stable integration into the surrounding corneal host tissue, epithelialization of the surface of the prostheses and corneal nerve regeneration [72]. Therefore, keratoprostheses are continuously optimized, e.g., by functionalizing with cell adhesion peptides [76] or growth factors [77]. 

## 2. Tissue Engineering of the Corneal Endothelium

### 2.1. General Considerations

Besides the aforementioned synthetic keratoprostheses, the use of biocompatible materials and cell-based tissue equivalents based on tissue-engineering strategies represents a further important therapeutic approach [78]. Like the native cornea, an artificial cornea has to integrate into the recipient tissue [79], support the formation of an intact epithelium and tear film and allow corneal innervation. These materials should not provoke complications, such as immunologic reactions, infection, glaucoma, formation of retroprosthetic membranes or device extrusions. Artificially generated corneal tissues need to adopt further corneal functions, like optical refraction, and should be composed of biocompatible materials that can be transplanted with up-to-date surgical techniques.

In principle, two tissue replacement strategies can be distinguished. One strategy aims at exclusive application of cells. For example, several groups transplanted a suspension of primary HCEC onto human and porcine corneas *in vitro* [80,81,82]. In this context, it was observed, that the morphology and cell density of the newly formed HCEC monolayer depended on the differentiation status of the transplanted primary HCEC and is influenced by the cell isolation and cell cultivation techniques used before transplantation [82,83,84]. For example, studies on transplantation of HCEC suspensions on de-endothelialized corneas *in vitro* showed that sufficient cell densities have been achieved when immortalized cell lines were used, but not with normal human cells [82,85]. Similar experiments carried out with animal-derived corneal endothelial cells, mostly from rabbit, showed better results regarding achieved cell densities. However, with the exception of cats, animal-derived corneal endothelial cells generally have a higher proliferative and, also, regenerative capacity than HCEC, which aggravates implementation of such studies into a clinically applicable technique [86]. Another method is based on incorporation of superparamagnetic microspheres into HCEC and the generation of an endothelial monolayer by placing a magnet in front of the donor cornea after injecting the cells as suspension into the anterior chamber [87]. Moreover, human cornea equivalents were created by controlled assembly of single cell layers composed of immortalized HCEC, native stromal cells (fibroblasts) or immortalized corneal epithelial cells using hanging cell culture inserts [88,89]. These cornea equivalents were designed for pharmaceutical studies and were shown to be similar to native human corneas with respect to their morphology and permeation behavior of conventionally applied ophthalmic agents. Unfortunately, the stiffness, curvature and transparency of naturally grown corneas could not be emulated with this method, so that the cornea equivalents are not suitable for transplantation. 

The second tissue replacement strategy focuses on biomaterial-supported, cell-based reconstruction of diseased corneal layers, with biomaterials serving as carriers and scaffolds for cells. The spectrum of these carriers and scaffolds includes naturally grown membranes, biological polymers and biosynthetic material composites, as well as completely synthetic materials. Various concepts for a carrier-based engineering of the corneal endothelium are presented in the following chapters.

### 2.2. Naturally Grown Membranes

Amniotic membrane, though not an ocular tissue, is used routinely to support wound healing after severe injuries of the ocular surface, because this membrane has strong anti-inflammatory, anti-angiogenic and wound healing supporting characteristics [90]. Besides therapeutic application, amniotic membrane was also successfully used as a carrier for *in vitro* cultivation of corneal endothelial cells [91,92]. Cultivation of the corneal endothelial cell line, IHCEn, on cell culture carriers composed of a lyophilized human amniotic membrane, which was installed on a Teflon ring, led to an enhanced expression of typical cell markers, compared to IHCEn grown on conventional tissue culture polystyrene [93]. In another study, cultivation of primary HCEC on Descemet’s membrane as the natural basement membrane of the corneal endothelium was analyzed [94]. It was demonstrated that pathologically changed Descemet’s membranes, like in the case of Fuchs’ endothelial dystrophy, impaired the growth of seeded HCEC. Furthermore, the suitability of anterior lens capsule as a carrier for cultivation of HCEC was investigated. The lens capsule enabled the formation of a confluent monolayer with a typical endothelial cell density, morphology and expression of typical cell markers [95]. Moreover, decellularized human corneal stroma [78] and decellularized porcine corneas [96,97] have been used as scaffolds to generate so-called “neo-corneas”, which were comparable to native corneas with respect to the morphology of seeded primary HCEC and their biomechanical properties. Finally, decellularized, bovine posterior corneal lamellae have been successfully applied as carriers for HCEC [98].

### 2.3. Biological Polymers

Besides naturally grown membranes, artificial membranes or scaffolds made of biological polymers were used as carriers for corneal tissue engineering. While naturally grown membranes harbor the risk of contamination with potentially infective substances or undefined and probably unwanted biological activity, due to cytokine deposition, carriers made from biological polymers have the advantage that they are of well-defined composition, while still biologically interacting with the cells. For example, gelatin, a denatured form of mostly collagen type I, was first utilized in 1980, where it was employed as a 1 µm thick, cross-linked gel carrier for cultivation of rabbit corneal endothelial cells [99,100,101]. After successful transplantation of these constructs into rabbit eyes *in vivo*, previously opacified corneas became clear again. Cultivation of primary HCEC on gelatin gels, which were coated with collagen type IV, resulted in the formation of confluent monolayers of regularly arranged, polygonal cells that produced the cell markers, ZO-1, Na^+^/K^+^-ATPase and N-Cadherin [102]. Similarly, gels of cross-linked, vitrified collagen type I (“Vitrigel”) were used to culture primate corneal endothelial cells. Transplantation of these constructs *in vivo* resulted in the long-term clarification of previously opacified corneas [103]. Transplantation of primary HCEC cultured on a network of loosely cross-linked collagen type I sheets *in vivo* into rabbit [104] and rat eyes [81] led to a recovery of corneal transparency in previously opacified corneas. Moreover, permeable and transparent membranes made of silk fibroin were applied successfully for cultivation of corneal endothelial cells [105]. The human corneal endothelial cell line, HCEC-B4G12 [106], was grown on such silk fibroin membranes after coating the membranes with various extracellular matrix (ECM)-components [107]. Only collagen type IV-coated silk fibroin membranes allowed for the formation of confluent monolayers, which were comparable to layers formed on non-coated tissue culture polystyrene (TCP). Based on these results, confluent monolayers of primary HCEC with a polygonal cell morphology could be established on collagen-coated silk fibroin membranes [107].

### 2.4. Composites Made from Biological and Synthetic Polymers

In addition to generating single-layered transplants, a scaffold comprised of collagen and chondroitin sulfate cross-linked with glutaraldehyde was used to generate whole corneal equivalents with all three corneal cell types [108]. The scaffold was invaded by immortalized stromal cells and seeded with immortalized corneal epithelial and endothelial cells. These corneal equivalents were comparable to native corneas with respect to their morphology, expression of cell-type specific proteins, transparency and transport of ions and fluids. As further developments of this approach, porcine collagen type I [109] and recombinant human collagen type I and III [110], which were cross-linked with 1-ethyl-3-(3-dimethylaminopropyl)carbodiimide (EDC) and N-hydroxysuccinimide (NHS), were used to generate scaffolds for corneal tissue engineering. A scaffold composed of collagen and poly(*N*-isopropylacrylamide-*co*-acrylic acid-*co*-acryloxysuccinimide) enabled the establishment of a corneal construct with improved mechanical properties. Additional functionalization with the laminin-derived peptide of tyrosine-isoleucine-glycine-serine-arginine (YIGSR) resulted in an increased innervation of these constructs [111]. In another approach, a collagen-sponge, made from a porous matrix of bovine, fibrillar collagen type I, was used as a scaffold for separate cultivation of human corneal keratocytes, epithelial and endothelial cells, as well as for co-cultivation of epithelial cells with keratocytes or epithelial with endothelial cells [112]. Using this system, it was shown that the thickness of the epithelial multilayer was influenced by soluble factors that were secreted by the endothelium and that keratocytes had an influence on the morphology of basal epithelial cells. Moreover, membranes based on chitosan were analyzed as possible carriers for corneal endothelial cells. Confluent cell layers of rabbit corneal endothelial cells were grown on polymer blends of hydroxypropyl- and hydroxyethyl-chitosan, gelatin and chondroitin sulfate [113,114]. After implantation into rabbits, these constructs induced only minimal inflammatory reactions and supported restoration of corneal transparency of previously opacified corneas. Similarly, bovine corneal endothelial cells were cultured successfully as confluent monolayers on polymer coatings comprised of chitosan and poly(ε-caprolactone) [115]. 

### 2.5. Synthetic Materials

Further advances in tissue engineering of corneal layers, especially of corneal endothelium, were made with synthetic materials, because these materials can be produced with a fully defined composition and designed features and structures. In first attempts, soft hydrogel contact lenses that were coated with a mixture of ECM-proteins were used as carriers in tissue engineering of the human corneal endothelium [116]. Corneal endothelial cells isolated from cats and rabbits established confluent monolayers on these carriers, thereby generating transplantable constructs composed of a carrier and cells. When these constructs were transplanted onto opacified corneas of cats and rabbits *in vivo*, corneal transparency could be restored. Examples of synthetic materials are biodegradable polymers, such as poly(L-lactic acid) and poly(DL-lactin-*co*-glycolic acid) [117]. Finally, coatings of poly(vinylidene fluoride) that were covered with collagen type IV allowed for the formation of confluent monolayers of primary bovine corneal endothelial cells with positive expression of typical cell markers [118].

### 2.6. Special Focus: Thermo-Responsive Polymers as Cell Culture Carriers for Corneal Endothelial Cells

#### 2.6.1. General Requirements and Working Principle

Stimuli-responsive polymers (SRP) are also known as “smart” polymers. These so-called “environmentally sensitive” polymers show rapid changes in their microstructure activated by changes in the environment. External stimuli can be temperature, pH, ionic strength, magnetic and electric fields, light, ultrasound and chemical species. The resulting macroscopic changes that occur are reversible, and the system returns to its initial state when the stimulus is removed.

For cell culture applications, temperature as an external stimulus is preferred [119]. This allows using the stimulus at physiological conditions [120]. Thermo-responsive polymer-based cell culture carriers allow the detachment of cells without proteolytic enzymes, like trypsin, or mechanical procedures, like scraping, which has the advantage of keeping all cell-cell and cell-matrix contacts intact. The benefit thereby is that the ECM of the cell layer stays in place around the cells, so cell viability and re-adhesion is enhanced [121,122,123,124,125]. 

**Figure 2:**
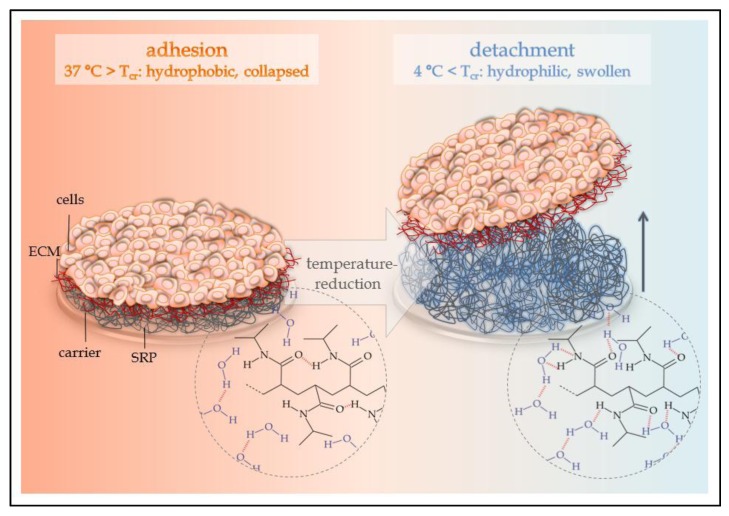
Function of thermo-responsive cell culture carriers based on poly(*N*-isopropyl acrylamide). (**Left**) At temperatures above the critical temperature, T_cr_, of the polymer system, intramolecular interactions between the single polymer chains are preferred. The polymer system is collapsed and allows for adhesion of cells. (**Right**) Reduction of the temperature beneath T_cr_ of the polymer system supports intermolecular interactions via hydrogen bonds between polymer chains and water molecules. The polymer system swells and thermally induced detachment of adherent cells as a sheet occurs. ECM = extracellular matrix; SRP = stimuli-responsive polymer.

Thermo-responsive polymers exhibit a critical phase transition temperature (T_cr_) due to the changes in the interactions between solvent (water) and polymer [126]. Below T_cr_, the polymer is fully hydrated (hydrophilic), but upon heating over T_cr_, the system ends up in phase separation (hydrophobic) [120]. The raised temperature results in a negative free enthalpy, which leads to a discrimination of the intermolecular hydrogen bonds between polymer and water. Intramolecular hydrogen bonds are preferred, and so, the polymer dehydrates [127], see Figure 2. 

The mechanism of adhesion and detachment of cells to or from thermo-responsive cell culture carriers has been widely investigated [128,129,130,131,132]. The adhesion of cells above the T_cr_ (hydrophobic state) is a complex combination of physicochemical effects, like hydrophobic and electrostatic interactions, as well as van der Waals forces. After the initial adhesion and the creation of focal adhesions, the reorganization of the cytoskeleton leads to spreading of the cell on the hydrophobic surface. The balance between initial cell adhesion, establishment of a confluent and functional cell monolayer and the stimulated detachment is quite hard to achieve.

To improve initial cell adhesion, the surface of thermo-responsive cell culture carriers can be coated by physisorption with ECM components, like collagens, fibronectin, laminins or other adhesion promoters based on peptides [133,134]. Another method is the direct modification of functional polymers, for example, by terminal carboxylation of the thermo-responsive polymer itself, which leads to an improved cell adhesion [135]. Furthermore, reactive copolymers can be introduced to tune the initial adhesion by providing covalently bound bioactive molecules, like the above-mentioned adhesion promoters [136]. 

After the cells reached confluence, the monolayer can be detached by reducing the temperature below T_cr_, thus resulting in the rehydration of the polymer network. The hydration of the polymer chains interrupts the interaction of the ECM molecules with the substrate. This abolishes the equilibrium of the tensile forces between the ECM and the cytoskeleton, which leads to a detachment of ECM from the hydrophilic surface and the final detachment of the whole cell layer, including the ECM. Optimal temperatures for the detachment vary between the different cell types of the tissue [125]. Usually, the cells are detached at room temperature, but 4 °C is also applicable. Specific ECM structures can inhibit the detachment from the hydrophilic thermo-responsive cell culture carrier. The application of hydrophilic comonomers within the thermo-responsive polymer can avoid such incidents [137]. Other criteria that influence successful detachment are the used cell culture media [129], the thickness of the thermo-responsive polymer layer [125,138,139], the swelling kinetics of the polymer layer or the mechanical properties of the swollen hydrogel [140].

#### 2.6.2. Thermo-Responsive Polymer Materials

Among the most important thermo-responsive polymers for biomedical applications [141] are poly *N*-substituted acrylamides, like poly(*N*-isopropyl acrylamide) (PNiPAAm) [142], poly(vinyl methyl ether) (PVME) [143,144,145] or poly(*N*-vinyl caprolactam) [146]. Copolymers [147] or polymer blends can combine a thermo-responsive structure with another structure in order to tune specific material properties.

The phase transition temperature of 32 °C of PNiPAAm dissolved in water is only a few K below the physiological cell cultivation temperature of 37 °C. This makes PNiPAAm a popular choice among thermo-responsive polymers for biomedical applications in general and stimulated cell detachment, in particular. The use of PNiPAAm for cell cultivation and thermally stimulated, enzyme-free cell detachment was reported for the first time in the early 1990s [148]. In this work, TCP dishes were coated with a thin PNiPAAm layer. Upon temperature reduction to 15 °C, the PNiPAAm layer was dissolved, while the confluent cell sheet was detached. Later, electron beam irradiation of a NiPAAm monomer solution spread on TCP surfaces allowed for the fabrication of stable thermo-responsive layers covalently attached to the carrier material [149]. Over the next few decades, this technique was continuously developed and applied to a multitude of cell types [119,150,151], including corneal endothelial cells [152,153]. Today, TCP dishes with electron beam grafted NiPAAm are commercially available (UpCell by Nunc, Thermo Scientific). Beside electron beam grafting, plasma-based deposition techniques can also be used to fabricate PNiPAAm coated surfaces. Furthermore, through this approach, corneal endothelial cell sheets have been generated and successfully tested *in vivo* [154,155].

To further optimize the properties of thermo-responsive cell culture carriers, copolymers can be used instead of the NiPAAm homopolymer. Following this route, a copolymer of NiPAAm with 2‑carboxyisopropylacrylamide [156] was proposed for thermo-responsive cell culture carriers that possess similar hydrophobicity and cell attachment properties compared to the NiPAAm homopolymer above the phase transition temperature, but with improved hydrophilicity and cell detachment below the phase transition temperature. In addition, a copolymer of NiPAAm and diethylene glycol methyl ether methacrylate [157] was investigated. Compared to pure PNiPAAm, the phase transition temperature of the thermo-responsive coating was even closer to the physiological cell cultivation temperature. The ethylene glycol content improved the balance of initial cell adhesion and stimulated detachment. Functional corneal endothelial cell sheets, including their ECM were detached faster upon a smaller temperature drop [158]. 

Beyond PNiPAAm and NiPAAM containing copolymers, other polymers with particular advantages [141] can be used to fabricate thermo-responsive cell culture carriers. This was shown for PVME, a material with a phase transition temperature of 34 °C [136]. Another way to tune the properties of thermo-responsive cell culture carriers are polymer blends. For that purpose, a thin film of a thermo-responsive polymer mixed with another functional polymer is prepared on a suitable surface followed by cross-linking and immobilization. This has been previously already demonstrated for PVME coatings. The second blend component used in this approach introduced reactive anhydride sites that could subsequently be used for biomolecular functionalization. Different proteins and peptides, covalently attached to the thermo-responsive cell culture carrier, gave the ability to tune the balance between initial adhesion and stimulated detachment of HCEC [159]. 

#### 2.6.3. Preparation of Thermo-Responsive Cell Culture Carriers

To prepare a cell culture carrier, a layer of a thermo-responsive material has to be formed and immobilized on a solid surface, whether organic or inorganic and with or without reactive sites. The variety of fabrication processes can be assigned to two major cases: those starting off with a thermo-responsive *polymer* and those starting off with a *monomer*. In both cases, techniques with and without additional energy input can be distinguished (Table 1). Each particular approach has specific advantages and limitations in terms of technical feasibility and achievable layer properties. 

**Table 1:** Processes to fabricate thermo-responsive coatings.

Process based on	Polymer/copolymer/blend	Monomer unit(s)
without additional energy input	grafting to	grafting from
**with additional energy input**	simultaneous cross-linking and immobilization by ˗ electron irradiation ˗ low pressure plasma exposure	simultaneous polymerization and immobilization by ˗ UV irradiation ˗ gamma irradiation ˗ electron irradiation plasma polymerization

*Grafting to* and *grafting from* are techniques that work without additional energy input on reactive (functionalized) surfaces of any type, either organic or inorganic [160]. In the case of *grafting to*, end functionalized polymers are grafted to reactive sites on the surface. This was demonstrated for carboxyl-terminated PNiPAAm and amino groups [161]. In the *grafting from* approach, a monomer solution is applied to a reactive surface, which leads to a continuous growth of polymer chains. Using this technique, brush-like structures of PNiPAAm have been prepared [162]. The *grafting from* approach allows for immobilization of a higher amount of polymer compared to *grafting to*. An improved polymerization process can be achieved by controlled radical polymerization, such as atom transfer radical polymerization (ATRP) [163]. 

All other described processes work with additional energy input via different mechanisms. Contrasting to the aforementioned cases, a pre-functionalized surface is not required. Starting from a monomer solution that is spread on an organic surface, simultaneous polymerization and immobilization of thin films can be achieved with different types of energetic radiation. This was demonstrated for ultraviolet (UV) radiation, where stable PNiPAAm coatings were obtained by UV exposure of a NiPAAm solution containing a photoinitiator [164]. No initiator is necessary when electron beam irradiation is used for PNiPAAm film formation. This approach is especially common and was investigated in detail. A well-defined film thickness up to 40 nm can be obtained by adjusting the concentration of the monomer solution and the absorbed energy dose [138,160]. Besides PNiPAAm, also copolymer coatings can easily be fabricated by this technique. For that purpose, a mixed solution of NiPAAm and a second functional monomer is applied and treated via electron beam [156]. 

A completely different approach that starts from the monomer and requires additional energy input is plasma polymerization [165]. Here, the gaseous monomer is introduced into a low pressure plasma, upon which the molecules undergo numerous radical formation and fragmentation processes. Subsequently, the activated species form a cross-linked polymeric layer on a solid surface appropriately placed in the plasma apparatus. This concept was successfully applied to fabricate thermo-responsive coatings using NiPAAm [123,166] or *N*,*N*-diethylacrylamide [167] as precursor molecules. 

Starting from a solid polymer thin film that is prepared on an organic surface, e.g., by spin coating, simultaneous cross-linking and immobilization can be achieved by energetic radiation or plasma. This was utilized to prepare stable PVME coatings on TCP by electron beam irradiation [136]. Contrary to PNiPAAm, PVME exhibits a high affinity to electron beam cross-linking [168]. This offers the possibility to fabricate covalently attached thermo-responsive coatings on polymeric carriers with a broadly variable thickness ranging from a few nm to a few µm, a well-defined cross-linking degree and, consequently, a well-defined swelling behavior in aqueous media [136]. The technique can also be applied to polymer blends, as was previously shown for PVME and the alternating copolymer of vinyl methyl ether and maleic anhydride (PVME-blend-PVMEMA) [159]. Instead of electron beam irradiation, a low pressure plasma can be used for simultaneous cross-linking and immobilization of thin polymer films on organic surfaces [169]. This has been demonstrated with the copolymer of NiPAAm and diethylenglycol methyl ether methacrylate, poly(NiPAAm-co-DEGMA) [158,170]. However, compared to the electron beam approach, the plasma immobilization technique is limited to a film thickness of a few tens of nanometers only.

#### 2.6.4. Characterization of Thermo-Responsive Polymer Coatings

Ensuring and optimizing the proper function of a thermo-responsive coating in contact with an aqueous environment requires comprehensive knowledge on the system properties. In case of switchable cell culture carriers, this includes not only chemical composition of the coating, but also temperature-dependent change of film thickness, wettability, surface morphology, elastic modulus, protein adsorption and, finally, cell adhesion and detachment characteristics. Below, a range of advanced analytical techniques and selected applications for characterizing thermo-responsive cell culture carriers are summarized.

X-ray photoelectron spectroscopy (XPS) allows for the study of the atomic composition and chemical structure of a material surface. The high surface selectivity in the range of only a few nanometers makes XPS highly useful for analyzing thin coatings on solid surfaces, such as PNiPAAm grafts on TCP. For that reason, XPS is frequently used to characterize thermo-responsive cell culture carriers that were prepared by various techniques [122,139,166,171,172,173,174,175]. 

Time of flight secondary ion mass spectrometry (ToF-SIMS) is another extremely surface-selective tool used to investigate the chemical composition of materials. This technique is frequently used to study the structure of thermo-responsive coatings as prepared [176,177], as well as the coverage of cell culture carriers with proteins [178] or remaining ECM components after stimulated cell detachment [179]. ToF-SIMS can be operated in a dynamic mode to acquire depth profiles on the micron scale, and lateral imaging is possible, as well. However, compared to XPS, the quantification of results is more demanding. 

Furthermore, infrared spectroscopy has been applied to prove successful formation of thermo-responsive coatings [135,166,180,181,182,183,184]. A surface selectivity in the range of a few microns is achieved by attenuated total reflection. Typically, this is more than the layer thickness and allows one to estimate the amount of immobilized PNiPAAm per unit area [138,175]. 

A more precise determination of layer thickness in the dry state can be achieved with optical techniques, like spectroscopic ellipsometry. With a liquid cell, spectroscopic ellipsometry allows to study temperature-dependent swelling and collapsing behavior in aqueous media *in situ* [145,170,185]. A plot of the film thickness over temperature reveals the precise phase transition value, but also gives information about the switching characteristics of the surface immobilized film. Depending on the preparation technique, this may be different from the corresponding polymer in solution, for example, in the case of electron beam cross-linking. Additionally, the impact of modification strategies, like copolymerization [158] or blending [136], on the thermo-responsive switching behavior can easily be evaluated. In the case of laterally-structured thermo-responsive coatings, imaging ellipsometry can provide 3D representations of the swelling and collapsing pattern [186].

An important property of a thermo-responsive cell culture carrier is its temperature-dependent wetting behavior. Since the surface under investigation is swelling, water contact angle measurement is a challenge; however, conventional contact angle goniometry can successfully be applied [125]. To improve the significance of wetting measurements on such systems, the captive bubble technique can be applied [187]. 

Atomic force microscopy (AFM) was used to study the surface morphology of thermo-responsive coatings in the dry state, as well as in the swollen state [139,181,188]. Furthermore, AFM can provide a simple proof of the temperature-dependent swelling and, also, allows for estimating the degree of swelling [176]. AFM-based nanoindentation measurements are the key to determine the elastic modulus of the surface on the nanometer scale [159,189,190].

A quartz crystal microbalance with dissipation monitoring (QCM-D) works with an oscillating quartz crystal at its resonance frequency. Measuring the shift in resonance frequency caused by a thin film that is deposited on the quartz crystal allows for calculation of mass per unit area. In the case of hydrogel films immersed in an aqueous environment, QCM-D also senses the mass of water molecules that are bound to the hydrogel due to hydration. Monitoring the decay of the quartz oscillation when switching off AC excitation provides additional information on the viscoelastic properties of the hydrated polymer layer. These capabilities make QCM-D a highly advantageous tool to characterize the phase transition of thermo-responsive polymer coatings [191], including the dynamic response to temperature changes [192] and protein adsorption phenomena [193]. 

#### 2.6.5. Application of Thermo-Responsive Polymers for Corneal Endothelial Tissue Engineering

Thermo-responsive cell culture carriers have been successfully employed for tissue engineering of cellular composites for non-ocular applications and, also, for reconstruction of the ocular surface [119,121,124,127,150,174,194]. In principle, cells are seeded as a suspension on the carrier and cultured at 37 °C to obtain a monolayered or multilayered cohesive, cellular composite. Importantly, essential functional characteristics have to be retained, preferably by applying optimal cell culture conditions. Upon lowering the temperature, the cells can be released from the carrier as a composite. They can then be transferred *in vitro* onto other artificial carriers for further analyses of morphology and expression of characteristic markers or can be transferred *in vivo* to support regeneration of diseased recipient tissue for preclinical or clinical studies. In contrast to naturally grown membranes or biological polymers, thermo-responsive cell culture carriers allow for generation of complex mono- or multi-layered cellular composites that can be transferred without the carrier itself [119,174,195,196,197,198,199,200,201,202]. Especially for ocular applications, the size, shape and localization of the transplant in the patient’s eye can be defined more precisely using thermo-responsive carriers compared to direct application of cell suspensions [119]. 

When the cells are detaching from thermo-responsive carriers, they usually retain their ECM, which can facilitate adhesion and integration of the cellular transplant at the target location and may render sutures unnecessary [203]. It was demonstrated that primary HCEC could be successfully cultured on electron beam grafted PNiPAAm-based cell culture carriers. These PNiPAAm carriers did not derogate cell viability [152,153]. Alternatively, primary HCEC could be cultured on PNiPAAm-based cell culture carriers that were generated by low pressure plasma-based deposition [155,204]. Stimulated detachment of HCEC monolayers at 20 °C did not alter or affect the morphology and viability of the cells. They still maintained their typical, hexagonal morphology with microvilli at their apical cell surface, showed immunopositivity for the tight junction protein, ZO-1, and for Na^+^/K^+^-ATPase at their lateral cell borders and retained their ECM. Nevertheless, it was also observed that detached monolayers had gaps between cells, which were ascribed to friction and traction within the cell layer during the detachment process. In another approach, a thermo-responsive carrier based on poly(NiPAAm-co-DEGMA) was prepared by low pressure plasma immobilization and was successfully employed to culture the HCEC cell lines, HCEC-12 and HCEC-B4G12, under serum-containing and serum-free conditions [158,205]. On this new material, both cell lines could establish confluent monolayers that were positive for ZO-1 and Na^+^/K^+^-ATPase at their lateral cell borders. Using fluorescently labeled fibronectin, it could further be demonstrated that matrix proteins were detached together with the cell sheet after thermal induction. 

The applicability of electron beam-grafted PNiPAAm-based cell culture carriers was further proven after *in vivo* transplantation of cultured primary HCEC into rabbit eyes [153]. Transplantation of the generated HCEC monolayers led to a significant decrease in the corneal thickness of rabbit eyes that were de-endothelialized before transplantation. For that matter, it was realized that the intraocular transfer of the fragile cell sheet was a great challenge [204]. In previous experiments using corneal epithelial cells, it was demonstrated that an extraocular application could easily be achieved with a circular poly(vinylidene fluoride) membrane, which was used as a stamp to stabilize the cell sheet during transfer. Thus, the cell sheet could adhere to the anterior corneal stroma, while maintaining correct cell polarization and orientation [206]. 

For both, a tissue-engineered endothelial transplant and a lamellar corneal endothelial donor transplant, intraocular transfer and stable adhesion at the posterior cornea are difficult to achieve. Minimally invasive surgical procedures, such as incisions of only a few millimeters in size, and the anterior chamber filled with aqueous humor are additional obstacles. The first attempts to improve intraocular transfer techniques focused on using a gelatin disk as a stable, biodegradable and adhesion-mediating tool [207]. This disk allowed for placing of an endothelial cell sheet on Descemet’s membrane of de-endothelialized rabbit corneas *in vivo* with subsequent restoration of corneal transparency. A further development of this transfer tool is a porous gelatin carrier, which can facilitate diffusion of nutrients from the aqueous humor to the grafted endothelial cell sheet [208,209]. In addition, the use of such gelatin carriers supports a correct apicobasal orientation of the cells during and after intraocular transfer.

**Figure 3:**
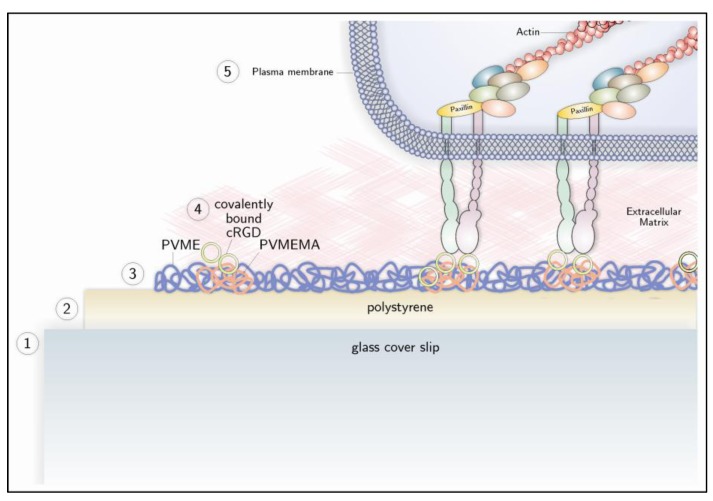
Preparation and biomolecular functionalization of a thermo-responsive cell culture carrier (**bottom**) and a cell attaching to it (**top**). A glass slide (1) is coated with polystyrene (2). A thin film of (PVME)-blend-PVME maleic anhydride (MA) (PVME-blend-PVMEMA) is prepared on the polystyrene surface by simultaneous electron beam cross-linking and immobilization (3). Anhydride groups allow for covalent attachment of proteins or peptides that contain free amino groups, like cyclic RGD (4). Subsequently, cells can attach to the surface by binding to the peptide with integrin receptors, which leads to the formation of focal adhesions at the intracellular side of the plasma membrane (5). Reprinted from [159] with permission from Elsevier.

**Figure 4:**
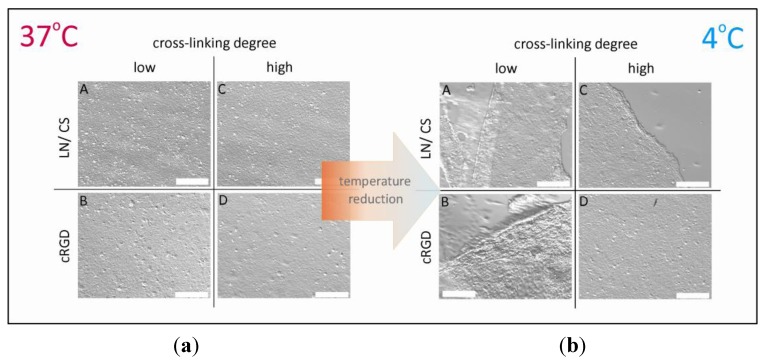
Selected cases of (**a**) adhesion; and (**b**) thermally stimulated detachment of human corneal endothelial cells (HCEC). Cells were cultured for four days on thin PVME-blend-PVMEMA-based carriers with a low content of reactive binding sites for proteins/peptides, a low/high cross-linking degree and biofunctionalization with laminin/chondroitin-6-sulfate (LN/CS) or cyclic RGD (cRGD).

To improve the balance between initial cell adhesion and stimulated cell sheet detachment, the physicochemical and biomolecular key properties of a thermo-responsive coating, such as thickness, stiffness, switching amplitude and biomolecular functionalization, can be adjusted in order to meet the specific requirements of corneal endothelial cells. Towards this goal, thermo-responsive cell culture carriers were fabricated by simultaneous electron beam cross-linking and immobilization of PVME-blend-PVMEMA (Figure 3). This allows for adjusting thickness and stiffness, as well as the swelling behavior of the thermo-responsive coating over a wide range. Furthermore, incorporation of the anhydride-containing blend component PVMEMA provides a well-defined density of binding sites for biomolecular functionalization without impairing the thermo-responsive properties [136]. This type of cell culture carrier was successfully employed to generate transferable cell sheets. It was shown that HCEC-12 formed monolayers with a regular morphology and high metabolic activity and had ZO-1 localized at the lateral cell borders, focal adhesions and deposition of ECM. Initial attachment of HCEC-12 was superior on carriers with high stiffness (a high degree of cross-linking) that were biofunctionalized with cyclic RGD (cRGD), while detachment was facilitated by lower stiffness (low degree of cross-linking) and biofunctionalization with laminin and chondroitin-6-sulfate (Figure 4) [159].

## 3. Conclusions

HCEC are non-regenerative *in vivo*; therefore, damage to the endothelial layer cannot be repaired by the cells themselves. Endothelial dystrophies can lead to corneal blindness and, consequently, can only be treated by keratoplasty. The shortage of corneal donor tissue limits conventional therapies that rely on full-thickness or lamellar corneal transplants. The ability of HCEC to proliferate *in vitro*, where they are strongly adherent, offers the possibility to develop alternative therapeutic strategies, such as tissue engineering approaches. These techniques are based on culturing the cells on a range of carriers, including naturally grown membranes, artificial membranes made of biological polymers or fully synthetic materials. Naturally grown membranes and artificial membranes made of biological polymers have the advantage of being highly biocompatible. However, all these materials are usually an integral part of the graft and, therefore, need to be transplanted together with the cells. Therefore, the additional material between the corneal endothelial cells and the stroma might create a diffusion barrier, which could lead to an impairment of the pumping function of the corneal endothelium and, thus, to a disturbance in the corneal hydration balance. Moreover, naturally grown membranes can harbor a cocktail of growth factors and cytokines, which may not only stimulate cell viability, but may also provoke immune reactions. Furthermore, some materials may induce inflammatory reactions while being resorbed by recipient cells. In this situation, thermo-responsive cell culture carriers offer the advantage that the carrier itself is not a part of the graft. Rather, the graft consists of only the cells and their ECM, which are detached as a contiguous sheet upon lowering the temperature. Thus, the risks of inducing undesired systemic reactions or inflammation due to cytokines or other bioactive substances or the impairment of the hydration balance due to the additional diffusion barriers are greatly minimized. Furthermore, the ECM transferred together with the detached HCEC represents components of Descemet’s membrane and might support cell sheet adhesion to the stromal bed. Besides the NiPAAm homopolymer—the most common material for this purpose—more sophisticated material compositions were recently developed. This includes copolymers and blends with NiPAAm or vinyl methyl ether as the responsive unit. A common goal of these approaches is to tune physicochemical and/or biomolecular interactions of the thermo-responsive carrier to meet specific needs of various cell types. The authors consider the application of thermo-responsive carriers for the engineering of corneal endothelial tissue highly promising, as it probably poses less risk of inducing complications than other carrier-based approaches. However, with all the advances in the field of carrier materials, the success of tissue engineering strategies for corneal endothelial replacement is hampered by two still unsolved problems that are related to corneal endothelial cell properties and current surgical techniques. Firstly, only donor primary human cells can be used for a clinical application, but their limited proliferative capacity impedes implementation of protocols developed in *in vitro* studies into a clinical application. Secondly, the transfer of a fragile, monolayered cell sheet into the anterior chamber and its stable fixation to the posterior cornea remains a surgical challenge that has not yet been met.
